# Colonization Resistance in the Infant Gut: The Role of *B. infantis* in Reducing pH and Preventing Pathogen Growth

**DOI:** 10.3390/ht9020007

**Published:** 2020-03-27

**Authors:** Rebbeca M. Duar, David Kyle, Giorgio Casaburi

**Affiliations:** Evolve BioSystems, Inc., Davis, CA 95618, USA; rduar@evolvebiosystems.com (R.M.D.); dkyle@evolvebiosystems.com (D.K.)

**Keywords:** fecal pH, infant microbiome, probiotics

## Abstract

Over the past century, there has been a steady increase in the stool pH of infants from industrialized countries. Analysis of historical data revealed a strong association between abundance of *Bifidobacterium* in the gut microbiome of breasted infants and stool pH, suggesting that this taxon plays a key role in determining the pH in the gut. *Bifidobacterium longum* subsp. *infantis* is uniquely equipped to metabolize human milk oligosaccharides (HMO) from breastmilk into acidic end products, mainly lactate and acetate. The presence of these acidic compounds in the infant gut is linked to a lower stool pH. Conversely, infants lacking *B. infantis* have a significantly higher stool pH, carry a higher abundance of potential pathogens and mucus-eroding bacteria in their gut microbiomes, and have signs of chronic enteric inflammation. This suggests the presence of *B. infantis* and low intestinal pH may be critical to maintaining a protective environment in the infant gut. Here, we summarize recent studies demonstrating that feeding *B. infantis* EVC001 to breastfed infants results in significantly lower fecal pH compared to controls and propose that low pH is one critical factor in preventing the invasion and overgrowth of harmful bacteria in the infant gut, a process known as colonization resistance.

## 1. Introduction

The healthy breastfed infant gut microbiome in the early 1900’s was reported to be dominated by a group of Gram-positive bacteria that today are recognized as *Bifidobacterium*. Particularly, in 1913, the stool of healthy newborn breastfed infants was described and documented as “films of almost pure culture”. Conversely, *Bifidobacterium* in the stool of infants fed cow’s milk were much lower in abundance and the stool was instead dominated by Gram-negative bacteria [1]. By 1926, the pH of the healthy newborn breastfed infant stool in the US was reported to have a mean of 4.88, but in infants who were not fed breast milk the stool pH was closer to 6.0 [2]. More than 20 years later, in 1955, the pH of breastfed infant stool was found to be between 5.3 and 5.5 [3]. Today, reports indicate the typical stool pH of infants, regardless of feeding type, to be as high as 6.5, corresponding to an increase of almost two log units from 1926 [4]. Together these records indicate the stool pH of infants has increased substantially during the last century, a period that corresponds with decreased rates of breastfeeding and a reduction in the abundance of *Bifidobacterium* [4]. It is worth noting that despite significant technological advancements during the past century, the analytical methods used to measure pH have remained largely unchanged, thus allowing direct comparisons of historical and contemporary reports. 

Interestingly, changes in infant stool pH appeared to have occurred predominantly in industrialized regions such as the United States and Europe [5]. Conversely, in less-industrialized nations, where breastfeeding rates remain high and microbiome-modifying interventions are less common (e.g., limited exposure to antibiotics and c-section births), *Bifidobacterium* dominates the microbiome of infants [6,7]. Most notably, in these nations the dominant *Bifidobacterium* species is *B. longum* subsp. *infantis* (*B. infantis*), which is conspicuously absent in industrialized nations [8,9,10]. In the United States, the microbiome of babies born today are not dominated by *Bifidobacterium*, and most notably is the lack of *B. infantis*, specifically [11,12]. Consequently, important ecosystem services of the microbiome are lost, which in turn leads to low community stability and diminished colonization resistance, which together are signatures of infant gut dysbiosis [13]. This species-specific loss of *B. infantis* from industrialized regions has been posed to be, in part, a consequence of its elimination from the mother’s gut microbiome through repeated use of antibiotics and other interventions known to have decimating collateral effects on the microbiome [14]. Thus, if the mother no longer carries *B. infantis* in her gut, there is no opportunity of transfer to the baby during the birthing process, which is the main path by which the newborns acquire their gut microbiome [15,16]. Overall, data points to a generational loss of *B. infantis* over the past century, a period that has also seen a significant rise in autoimmune and allergic diseases [17,18]

## 2. Lowering pH is a Biological Strategy to Limit Bacterial Growth

The pH of a microbial ecosystem is heavily defined by the metabolites produced by the inhabitants of that ecosystem. Most opportunistic bacterial pathogens prefer to grow at pH approaching neutrality (pH = 6.0–7.0) and grow poorly in acidic conditions (pH ≤ 5.5). Lactic acid bacteria (e.g., *Bifidobacterium* and *Lactobacillus*) produce acetic and lactic acids as major metabolic end products, which in turn significantly lowers the luminal pH [19]. Lactic acid bacteria have been used in preservation of certain foods for millennia [20], as it prevents the growth of spoilage and pathogenic organisms. This same biological control system is also at work in our bodies. For example, the vaginal mucosa of reproductive-age women is generally dominated by lactic-acid producing bacteria such as *Lactobacillus* spp. [21] and the healthy vaginal environment is distinctly acidic, with a pH of 4.5–5.5 [22]. A change to an elevated pH is often associated with bacterial vaginosis and the overgrowth of opportunistic pathogenic fungi [21,23]. Although additional hormonal and physiological factors are involved in protecting the vaginal environment, the pH has an important role in protecting the vagina from pathogens [24]. This natural prevention of pathogen invasion, through lowering the pH and exemplified by acidification of the vaginal environment, is one important strategy by which bacterial communities that inhabit the gut protects the host from invading pathogens and is a process known as colonization resistance [25,26].

## 3. Newborns in the US May Have Reduced Pathogen Colonization Resistance

One of the most important roles of the gut microbiome is to provide colonization resistance from invading intestinal pathogens throughout life [26]. However, this protective role becomes especially important during the neonatal period when the immune system is immature [13,27]. The gut microbiome of the infants born in industrialized nations today is not dominated by *Bifidobacterium* and is missing *B. infantis*. Therefore, colonization resistance provided by acidification of the lumen through the production of lactate and acetate has been lost, or at least reduced [10,11,12,28]. The microbiomes of infants born today in most industrialized nations present signatures of low community stability and carry a high abundance of potential pathogens in the first months of life [29,30]. In clinical trials where *B. infantis* EVC001 was fed to breastfed infants, the mean fecal pH was reduced to 5.15 ± SD 0.42, the gut microbiome was dominated by *Bifidobacterium*, and the populations of opportunistic pathogens were significantly reduced [11,31].

The mechanism by which *B. infantis* becomes dominant in the gut and reduces pH is by its unique ability to metabolize all human milk oligosaccharides (HMO) into acidic end products, mainly lactate and acetate. HMO make up to 15% of the energy content of human milk and over 200 distinct complex HMO structures have been identified. HMO in human milk cannot be metabolized by the infant or by the vast majority of bacteria that inhabit the infant gut which lack the required enzymes necessary to access and metabolize these complex glycan molecules [32]. However, *B. infantis* is the only known organism capable of consuming all HMO structures in mother’s milk due to its unique genetic architecture. Specifically, *B. infantis* contains clusters of unique genes that encode for proteins required for the capture, uptake, deconstruction and metabolism of the HMO in human milk [33]. Consequently, we and others [34] posit that human milk is a selective growth medium for *B. infantis*, the engraftment of which, establishes colonization resistance in the infant gut by lowering the pH [13].

**Figure 1:**
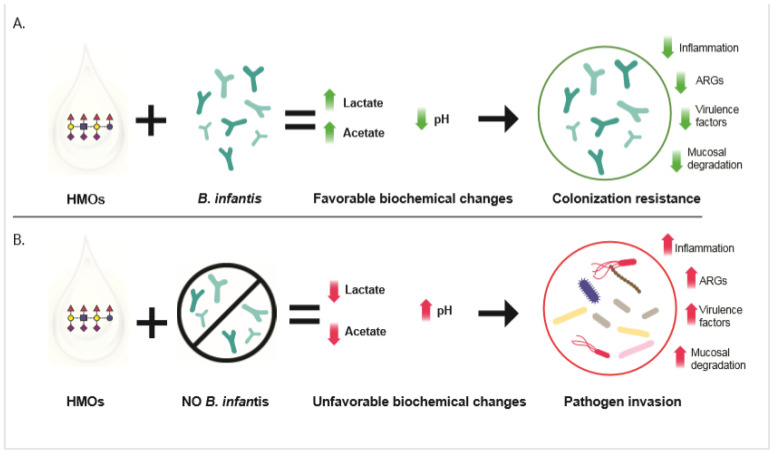
The breastfed infant gut environment resulting from the presence (**A**) or absence (**B**) of *B. infantis*. (**A**) In the presence of *B. infantis,* human milk oligosaccharides (HMO) from breastmilk are efficiently metabolized and converted in metabolic acidic end products, mainly acetate and lactate. These compounds lower the intestinal pH providing colonization resistance. (**B**) In the absence of *B. infantis*, HMO are not entirely metabolized and are lost in frequent stooling. The intestinal pH remains at high levels that permit pathogen invasion and replication resulting in both acute and long-term negative health outcomes.

In the absence of *B. infantis*, the HMO are almost completely eliminated in the stool as humans lack the enzymes required to metabolize them [11,35]. Consequently, if the infant gut microbiome is not producing high levels of acetate and lactate, the colonic pH remains high and it no longer provides colonization resistance to gut of the infant [11,13]. Cohort studies indicate the lack of colonization resistance and subsequent higher pH in the gut of the breastfed infants results in increased levels of potentially virulent pathogens [31], an increased antibiotic resistant gene load [12], and chronic enteric inflammation within the first 100 days of life [36], which has a strong association with increased prevalence of autoimmune disorders [37,38] (Figure 1).

## 4. Reestablishing *B. infantis* Restores Colonization Resistance to the Infant Gut

A controlled clinical intervention trial showed that when *B. infantis* EVC001 was fed to exclusively breastfed babies, their stool pH and stooling patterns returned to the condition reported in 1900’s (i.e., stool pH of 5.1 and 2–3 soft stools per day) compared to the control infants who produced 5–7 watery stools per day with a stool pH of 6.1 [5]. The control infants, which were all exclusively breastfed and mostly vaginally delivered, were missing *B. infantis* and presented a marked dysbiosis signature and high levels of potentially pathogenic bacteria [11]. 

Furthermore, 100% of the infants fed *B. infantis* EVC001 established a highly stable gut microbiome dominated by *B. infantis* (up to 90% relative abundance) [11,12]. Interestingly, while the dominance of *B. infantis* EVC001 had a substantial effect on microbiome composition in terms of relative abundance (beta diversity), there were no differences in terms of microbial species richness (alpha diversity). Specifically, we observed a similar Shannon diversity index in both controls and *B. infantis* EVC001-fed infants. However, the relative abundance of virulent pathogens such as *E. coli*, *Klebsiella*, *Clostridium* and *Staphylococcus* were all decreased by over 93% [31] and the bacterial antibiotic resistant gene load in the infant gut was also reduced by 90% relative to the control breastfed infants [12]. Further investigation indicated that control infants exhibited strong signals of erosion of the colonic mucous layer that protects the intestinal epithelium [39] and significant enteric inflammation in the first 40–60 days of life [36]. Conversely, infants that were colonized by *B. infantis* EVC001 had much less evidence of mucous layer erosion and inflammation over the same time period [36,39]. It is worth noting that *B. infantis* also contributes to maintaining the intestinal barrier integrity through the production of acetate and tryptophan compounds [40,41,42].

## 5. Low pH Is Key to Colonization Resistance in the Infant Gut

The vaginal microbiome and the infant gut microbiome are two examples of microbial ecosystems which are nominally dominated by lactic acid bacteria, and where the resulting lower pH provides a resistance to invasion by opportunistic pathogens. When these functional microbiomes are disrupted, colonization resistance is subsequently reduced, and the underlying tissues are put at risk [13] (Figure 1). The lack of colonization resistance in the newborn infant may be of particular concern since the first 100 days of life is a critical period for the development of the immune system [27,43]. Seventy percent of the immune system is associated with the gut [44] and in the first months of life patterns associated with the recognition of self and non-self are established with life-long consequences [27,30,43,45].

Intriguingly, along with the loss of colonization resistance in the infant gut over the past 100 years, there has been a dramatic increase of autoimmune disorders (e.g., asthma, atopic dermatitis, food allergies, Type I Diabetes, etc.) across industrialized nations including the United States [45,46]. Although it is tempting to make a causal link in this association, no randomized controlled trials have been undertaken as yet to establish whether the restoration of colonization resistance early in an infant’s life can deflect the development of autoimmune conditions later in life. However, increased biomarkers associated with the development of allergic and autoimmune conditions [37,38] and blunting of vaccine responses [6,8] in infants with microbiomes depleted in *Bifidobacterium*, suggests a plausible linkage between the reduced colonization resistance and a compromised development of the immune system [47].

## 6. Conclusions and Outlook

Altogether, data strongly indicate that the absence of *B. infantis* in the infant gut microbiome, and the associated loss of acetate and lactate production, leads to a stool pH above 5.5. This elevated stool pH decreases colonization resistance and allows the overgrowth of harmful bacterial populations [31,39] which are strongly linked to enteric inflammation [36] and an increased incidence of allergic and autoimmune disorders later in life [45]. When *B. infantis* EVC001 was reintroduced to the infant gut, fecal pH was reduced to levels that are inhibitory of pathogen growth and the natural colonization resistance to the infant gut was restored [11,13]. Given that aberrant gut microbiome compositions in early life (i.e., dysbiosis) are important determinants of acute and chronic ailments later in life [27,43], restoring *B. infantis* to the infant gut may be a key step towards improving lifetime health of future generations.

Furthermore, based on historical precedence and the present situation in populations from nations with limited exposure to gut microbiome-damaging practices such as excessive antibiotic use and c-section births, we propose that the reference range for a healthy infant fecal pH should be established between 4.5 and 5.5. Overall, research summarized here indicates that infant fecal pH above 5.5 is accompanied by a loss of colonization resistance as reflected by the increased abundance of potential pathogens, degradation of the gut mucosal barrier, and inflammation [13,36,39]. For this reason, fecal pH at or above this level should be classified as outside of a normal/adequate range. We recognize that some intestinal conditions such as those driven by carbohydrate malabsorption may also decrease the fecal pH [48]. However, due to the relative infrequency of such conditions versus the current high prevalence of dysbiosis-related elevated fecal pH in infants, we submit that the individual medical interest and the public health interest are best served by the proposed change.
